# Differences in the miRNA signatures of chronic musculoskeletal pain patients from neuropathic or nociceptive origins

**DOI:** 10.1371/journal.pone.0219311

**Published:** 2019-07-05

**Authors:** Camille Florine Dayer, François Luthi, Joane Le Carré, Philippe Vuistiner, Philippe Terrier, Charles Benaim, Jean-Paul Giacobino, Bertrand Léger

**Affiliations:** 1 Institute for Research in Rehabilitation, Clinique Romande de Réadaptation, Sion, Switzerland; 2 Department of Medical Research, Clinique Romande de Réadaptation, Sion, Switzerland; 3 Department of Musculoskeletal Rehabilitation, Clinique Romande de Réadaptation, Sion, Switzerland; 4 Department of Physical Medicine and Rehabilitation, Orthopedic Hospital, University Hospital of Lausanne, Lausanne, Switzerland; 5 Haute Ecole Arc Santé, HES-SO University of Applied Sciences and Arts Western Switzerland, Neuchâtel, Switzerland; Institut de Pharmacologie Moleculaire et Cellulaire, FRANCE

## Abstract

**Background:**

The quality of life for millions of people worldwide is affected by chronic pain. In addition to the effect of chronic pain on well-being, chronic pain has also been associated with poor health conditions and increased mortality. Due to its multifactorial origin, the classification of pain types remains challenging. MicroRNAs (miRNA) are small molecules that regulate gene expression. They are released into the bloodstream in a stable manner under normal and pathological conditions and have been described as potential biomarkers. In the present study, we aimed to investigate whether pain may induce an aberrant, specific dysregulation of miRNA expression, depending on the origin of the pain.

**Methods and findings:**

To do so, we measured the expression changes of 184 circulating miRNAs (c-miRNAs) in the plasma samples of patients with different origins of chronic musculoskeletal pain. After statistical analyses, we identified seven c-miRNA candidates that were differentially expressed depending on the nociceptive or neuropathic origin of the pain. We then developed a two c-miRNA signature (hsa-miR-320a and hsa-miR-98-5p) that was able to correctly classify the pain type of 70% of the patients from the validation set.

**Conclusions:**

In conclusion, circulating miRNAs are promising biomarkers to identify and characterize the chronic pain type and to further improve its clinical management.

## Introduction

Chronic pain is a major health care problem that affects millions of people worldwide (for review see [1]). In addition to its non-negligible effects on well-being and health costs, severe chronic pain has been strongly associated with a reduction in life expectancy, independent of sociodemographic factors [2]. The determination of chronic pain’s origin remains challenging and relies mainly on subjective rating systems, such as visual analogue scales, combined with thorough physical examination.

The criteria for the characterization of the different types of pain have profoundly evolved during the last decade, allowing the definition of 2 main pain families: nociceptive and neuropathic [3]. Nociceptive pain (No) is induced by the activation of the nociceptors either at the surface of the body (i.e., skin) or at a deeper level (i.e., muscles, bones, joints or tendon). It is particularly associated with arthritis but can also develop or increase in intensity after tissue injury. In this case, we speak about chronic posttraumatic pain. Chronic primary musculoskeletal pain syndromes are discriminated upon the duration of the symptoms (> 3 months) and upon the location: upper (chronic primary cervical pain), middle (chronic primary thoracic pain), lower back (chronic primary low-back pain), and limbs (chronic primary limb pain) [3].

Neuropathic pain (Np) is caused by an alteration or a disease of the central or peripheral nervous system. The pain may either appear spontaneously or in response to a stimulus. According to Scholz *et al*., neuropathic pain is also associated with hyperalgesia or allodynia [4]. A grading system for the diagnosis of Np has been proposed by Treede *et al*.[5], which relies on four criteria: (1) pain with a distinct neuroanatomical distribution; (2) a history suggestive of a relevant lesion or disease affecting the peripheral or central somatosensory system; (3) a demonstration of the distinct neuroanatomical plausible distribution by at least one confirmatory test (i.e., electrophysiology and biopsies); and (4) a demonstration of the relevant lesion or disease by at least one confirmatory test. The diagnosis of Np is “*possible*” if criteria 1 and 2 are met; “*probable*” if criteria 1 and 2 plus either 3 or 4 are met; and “*definite*” if all criteria are met.

The combination of neuropathic and nociceptive components (“mixed”) or the presence of inflammatory signs associated with chronic regional pain syndrome (CRPS) makes the diagnostic even more complex. Several epidemiological studies have demonstrated that most patients suffering from chronic neuropathic pain receive suboptimal treatment, mainly because of the lack of diagnostic accuracy [6, 7].

The treatment of chronic pain could greatly benefit from an accurate distinction between nociceptive or neuropathic pain. Due to this, it would be important to discover new biomarkers in easily accessible body fluids. Until now, most of the work performed on pain biomarkers has focused on secreted inflammatory mediators (i.e., cytokines). However, there has been no evidence that cytokines can be used as a relevant biomarker for diagnosing chronic pain [8].

MicroRNAs (miRNAs) are small fragments (< 23 nucleotides) of noncoding RNA that interact with messenger RNAs (mRNAs) to control protein translation. This direct interaction between miRNAs and mRNAs allows a thin layer of gene expression regulation at the posttranscriptional level. Since their discovery in the early 90s, miRNAs have been associated with many physiological processes [9–11]. To date, more than 2300 mature human miRNAs have been identified and are thought to contribute to the regulation of up to one-third of human coding genes [12, 13]. MiRNAs can be found in the bloodstream as all cell types release miRNAs under both normal and pathological conditions [14–17]. These c-miRNAs are either packed into 40–100 nm lipoprotein vesicles called exosomes or are bound to protein complexes [18, 19]. Although the possible function of released miRNAs remains largely unknown, some evidence strongly supports their role as regulators of the translation of target genes in recipient cells [20, 21]. The great stability of c-miRNAs in the plasma and serum also supports their potential as reliable biomarkers [22]. Therefore, expectations are high that the measurement of c-miRNAs might become a tool to improve the diagnosis and treatment of populations at risk and to improve the targeted prevention strategies [23].

Only a few studies have investigated changes in c-miRNA levels associated with painful diseases in humans. Orlova et al. reported changes in the levels of 4 c-miRNAs associated with CRPS [24]. Linnsteadt et al. [25] reported changes in the levels of 1 c-miRNA and Bjersing et al. [26] reported changes in the levels of 8 c-miRNAs associated with post-traumatic musculoskeletal pain or fibromyalgia. A recent study from Leinders et al. also demonstrated aberrant levels of 114 c-miRNAs associated with fibromyalgia [27]. Interestingly, the use of 5 miRNAs as signatures of fibromyalgia has already been suggested to enhance the potential of these molecules as diagnostic tools [28]. To date, no study has compared the changes in the c-miRNA profiles of different subtypes of chronic pain.

In the present study, we screened a large panel of circulating miRNAs in patients with chronic pain following traumatic skeletal muscle injury. The hypothesis guiding our investigations was that pain, depending on its category, may induce aberrant specific dysregulations of miRNA expression and/or release. To test this hypothesis, we compared the c-miRNA profiles in the two main subtypes of chronic pain, i.e., nociceptive and neuropathic.

## Materials and methods

### Study population

Patients with chronic musculoskeletal pain (CMSP) (lasting for more than 3 months) resulting from an orthopedic trauma were recruited between March 2014 and October 2017 from the Musculoskeletal Rehabilitation Department of the Clinique romande de réadaptation. The exclusion criteria were as follows: a history of diabetes, toxicomania, blood disease (i.e., thalassemia) and language barriers.

Immediately upon admission, the patients were thoroughly examined by a senior clinician to assess whether they met the inclusion criteria. On the basis of the DN4 questionnaire [29] and medical history, the patients were classified into one of the four groups of chronic pain (i.e., neuropathic, nociceptive, mixed or CRPS) according to the recommendations of the International Association for the Study of Pain (for details see:[4, 30]). For the profiling phase, 21 healthy controls, who reported no pain, were included for analysis. They were matched for age, sex and BMI with the 73 patients analyzed in the profiling phase (see Table 1). After they gave their written informed consent, the patients and controls were scheduled for blood sampling.

**Table 1:** The clinical characteristics of the participants in the profiling and validation set.

	Profiling set						Validation set		
Characteristics	Neuropathic (N = 15)	Nociceptive (N = 24)	Mixed (N = 17)	CRPS (N = 17)	Controls (N = 21)	p-value	Neuropathic (N = 40)	Nociceptive (N = 60)	p-value
**Age**	42 (15)	38 (2)	44 (13)	44 (10)	40 (10)	0.58	46 (12)	43 (12)	0.14
**Sex F**	3 (20%)	6 (25%)	5 (29%)	6 (35%)	7 (33%)	0.93	11 (28%)	7 (12%)	0.06
**BMI**	26 (2)	27 (5)	26 (5)	24 (5)	24 (3)	0.28	27 (4)	27 (4)	0.88
**Time since injury (days)**	705	593	500	357	-	0.50	532	472	0.48
**DN4 ≥ 4**	15 (100%)	3 (13%)	14 (82%)	10 (59%)	-	<10^−6^	38 (95%)	5 (8%)	<0.01
**Pain location**						0.30			0.83
**- Upper limb**	6 (40%)	8 (33%)	5 (29%)	10 (59%)	-		10 (25%)	20 (33%)	
**- Lower limb**	4 (27%)	9 (38%)	9 (53%)	7 (41%)	-		20 (50%)	28 (47%)	
**- Rachis**	4 (27%)	6 (25%)	2 (12%)	0 (0%)	-		8 (20%)	10 (17%)	
**- Polytrauma**	1 (7%)	1 (4%)	1 (6%)	0 (0%)	-		2 (5%)	2 (3%)	
**Medication (**patients taking any medication)	12 (80%)	13 (54%)	14 (82%)	15 (88%)	-	0.05	31 (78%)	47 (78%)	0.89
**Had a surgery**	12 (80%)	19 (79%)	14 (88%)	10 (59%)	-	0.25	31 (78%)	43 (73%)	0.46
**Pain (0–10)**	5.4 (1.9)	4.6 (2.0)	5.0 (1.8)	4.5 (2.0)	-	0.74	5.2 (2.1)	4.4 (2.0)	0.14
**Anxiety (0–21)**	10.1 (4.2)	9.0 (4.3)	8.5 (3.7)	11.2 (4.0)	-	0.31	9.9 (4.3)	8.6 (4.2)	0.15
**Depression (0–21)**	8.0 (4.9)	6.0 (3.5)	6.4 (3.2)	7.2 (3.2)	-	0.56	8.8 (4.2)	7.3 (3.9)	0.09
**TSK (17–68)**	43.7 (9.3)	45.4 (7.5)	41.2 (7.6)	43.2 (8.5)	-	0.31	44.4 (8.4)	43.6 (7.7)	0.70
**PCS (0–52)**	22.9 (11.7)	21.2 (10.3)	21.1 (11.0)	25.2 (9.9)	-	0.64	25.4 (11.8)	19.3 (11.6)	0.02
**CIRS (0–56)**	4.5 (1.7)	4.5 (2.4)	4.9 (2.7)	3.6 (2.0)	-	0.52	5.3 (3.2)	4.0 (2.2)	0.05
**CIRS active (comorbidities > 2)**	12 (80%)	20 (83%)	16 (94%)	12 (71%)		0.32	33 (83%)	40 (67%)	0.05

BMI, body mass index; DN4, Douleur Neuropathique en 4 Questions; TSK, Tampa Scale of Kinesiophobia; PCS, Pain Catastrophizing Scale; CIRS, Chronic Illness Resources Survey. The values are expressed as the mean (S.D.) or N (%).

Analyses were performed on 73 and 100 chronic musculoskeletal pain patients for the profiling and validation sets, respectively. An approximate number of patients per group was estimated from previous studies on miRNA detection [24, 31–33].

For each patient, the psychological status and pain levels were monitored using validated questionnaires, while the clinical information was collected from their medical records. Medication has been shown to influence miRNA expression [34]. However little is known about the influence of specific medicine on miRNA expression pattern. In a recent study, Almenar-Pérez et al. demonstrate the impact of polypharmacy on potential miRNA biomarker for the diagnosis of fibromyalgia [35]. To limit any possible influence from the medication in our study, the percentage of patients receiving medicine in the validation phase was adjusted between the No and Np groups.

The study protocol was approved by the local ethics committee (CCVEM 034/12) and was conducted according to the recommendations of the Declaration of Helsinki. Informed consent was collected from each patient involved in this study.

### Study design

**Fig 1:**
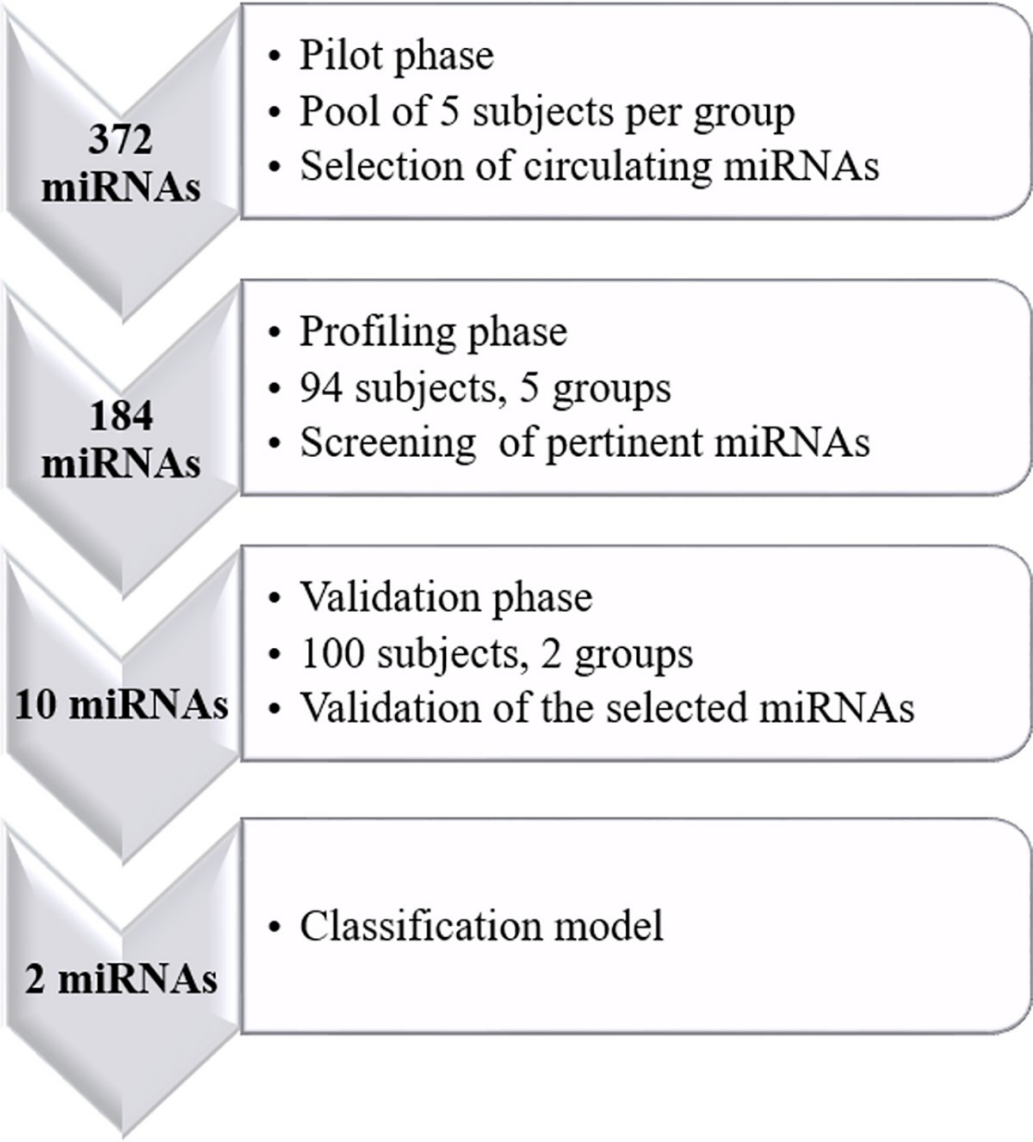
The study design scheme outlying the 3 different phases: Pilot, profiling and validation. Profiling and validation involved different groups of patients: for the profiling stage, Ctrl, No, Np, mixed and CRPS volunteers were recruited; in the validation stage, No and Np patients were enrolled.

Fig 1 represents the study design scheme that outlines the 3 different phases: pilot, profiling and validation. Initially, a pilot study was run with pooled samples to measure the expression of 372 miRNAs to determine that the miRNAs were accurately and reliably measurable in the plasma samples of our patients. Based on a protocol described elsewhere [36], five random RNA pools corresponding to each group were generated (five subjects per group), reverse transcribed and measured on the miRNA Ready-to-Use PCR Human panel I from Exiqon (372 human miRNAs). Panel I for humans contains the so-called high priority miRNAs that have been shown to be the most highly expressed, the most likely to be differentially regulated by diseases or the most cited in the literature [37].

For the profiling phase, 184 microRNAs were selected from the pilot study and were measured from the plasma samples of 73 patients and 21 controls distributed into 5 groups (24 No, 15 Np, 17 mixed, 17 CRPS and 21 controls) (S1 Table).

Finally, the validation step assessed the expression levels of 10 of the selected c-miRNAs in a population of 100 CMSP patients (60 No and 40 Np).

### Diagnostic criteria

The patients were classified into four different pain categories based on the following criteria:

- Nociceptive pain: pain that arises from damage to nonneural tissue and is due to the activation of nociceptors [38]. The term is used to describe pain occurring with a normally functioning nervous system to contrast with neuropathic pain [3]. In this study, the damage was secondary to the well-documented musculoskeletal injuries (medical records and images) after the orthopedic trauma (sprains, fractures…).
- Peripheral neuropathic pain: the patients classified as “probable” or “definite” according to the grading system for the diagnosis of Np described above here [5]. The DN4 (for « Douleur Neuropathique en 4 Questions ») questionnaire was used at the first step of this grading [29].
- Mixed pain: pain that arises from clinical situations combining nociceptive and neuropathic pain components. For instance, evidence of mixed pain in chronic low back pain or soft tissue injuries is present in the range of 28% to 71% of patients [39]. DN4 is applicable to detect this neuropathic component in a similar way as in neuropathic pain conditions [40]. In this study, mixed pain was retained on the basis of the DN4 and medical records.
- Complex Regional Pain Syndrome type 1 (CRPS type 1): persistent pain arising from CRPS is due to inflammation, vasomotor dysfunction and maladaptive neuroplasticity [41]. In this study, the CRPS diagnosis was retained according to the research Budapest criteria [42]. To avoid confusion with neuropathic pain conditions, patients with CRPS type 2 (with the established presence of a lesion of a peripheral nerve structure) were excluded.

The pain classification was based on a multidisciplinary assessment including therapists and physicians, with at least one senior physician. If there was any doubt about this categorization, the cases were reviewed to find a consensus with the patients’ senior registrars and, if necessary, with their neurologists and/or radiologists.

### Questionnaires

The DN4 questionnaire was developed to diagnose polyneuropathy. DN4 is a clinician-administered questionnaire. Seven items are related to pain symptoms, and three items are linked with neuropathic pain examination. Health professionals assess whether there is a reduced sensation to touch or a pinprick (hypoesthesia) and whether light brushing increases or causes pain (allodynia). The DN4 questionnaire has been broadly used and validated [40].

The Brief Pain Inventory (BPI) is a self-administered questionnaire that allows patients to rate the severity of their pain and the degree to which their pain interferes with the common components of feeling and function [43].

The Hospital Anxiety and Depression Scale (HADS) was used to detect states of anxiety and depression. This self-administered questionnaire has been specifically developed in a hospital medical outpatient clinical setting [44].

We used the Tampa Scale for Kinesiophobia (TSK: range 17–68) to assess pain-related fears (fears of movement and reinjury). Higher scores indicate more pain-related fears and were consistently related to higher pain and disability levels [45].

We used the Pain Catastrophizing Scale (PCS: range, 0–52) [46] to investigate catastrophic thinking (rumination, magnification and helplessness) that is a risk factor for pain chronicity.

The Cumulative Illness Rating Scale (CIRS) is a clinician-oriented instrument used to assess comorbidities and illness severity [47].

### Blood plasma collection and RNA isolation

Peripheral blood samples were collected in the morning (after an overnight fast) from the antecubital vein in 7.5 ml EDTA tubes (Sarstedt S-Monovette) using a butterfly device with a needle of 21 G. The tubes were immediately centrifuged at 3,000 g at 4°C for 10 min, the plasma was aliquoted in 1.5 ml RNase-free and DNase-free tubes and the samples were stored at -80°C until further processing. Only the plasma samples without visible signs of hemolysis were considered for the study [48].

Prior to RNA isolation, the plasma samples were centrifuged for 5 min at 3,000 g. Then, 300 μl of supernatant was transferred into a new tube for RNA extraction using the Exiqon miRCURY RNA isolation kit for biofluids (Exiqon, Vedbaek, Denmark), following the manufacturer’s instructions. The total RNA was eluted in 50 μl RNase-free water. Then, 1.5 μl (3 fmole) of UniSp2, UniSp4, UniSp5 RNA spike-in template mix (Exiqon, Vedbaek, Denmark) was added as a quality control to the lysis solution buffer before the RNA purification process.

### cDNA synthesis

All cDNAs from the profiling and validation step were reverse-transcribed using the Universal cDNA synthesis kit II from Exiqon according to the manufacturer’s protocol. For the profiling phase, 4 μl of RNA was added to the reverse transcription (RT) mix containing 4 μl 5x reaction buffer, 2 μl enzyme mix, 1 μl UniSp6 spike-in and 9 μl H_2_O.

For the validation phase, the miRNA concentrations were first assessed using the Quant-iT microRNA assay kit from Life Technologies following the manufacturer’s instructions. Briefly, 16 μl of plasma RNA was added to 200 μl of working solution, and the fluorescence was read on a microplate reader (Biotek Synergy HT Multi-Detection Microplate Reader, Biotek Instruments Inc.). All miRNAs were then diluted to 5 ng/μl, and 8 μl of the diluted miRNAs were reverse transcribed with minor modifications to the original protocol. The H_2_O volume was adjusted to 5 μl to obtain a final volume of 20 μl. We performed several tests to define the optimal volume of diluted miRNAs for RT.

### Quantitative PCR

For the profiling and validation phases, real-time qPCR was performed using the Pick-and-Mix microRNA PCR Panel from Exiqon. Just before the amplification, the cDNAs were diluted 50-fold in RNase-free water and were assayed with a 10 μl PCR according to the manufacturer’s instructions. For the validation study, all of the samples were measured in triplicate using the LightCycler 480 Real-Time PCR system (Roche, Rotkreuz, Switzerland) as previously described [49, 50].

### Data quality assessment

Quality controls for RNA isolation and cDNA synthesis were performed to check for differences in efficiencies and to identify experimental and technical outliers. UniSp2 and UniSp6, two synthetic miRNAs, were added during the RNA isolation and cDNA synthesis steps, respectively, as recommended by the Biofluids Guidelines from Exiqon. A coefficient of variation (CV) was calculated for UniSp2 and UniSp6 to determine the intrasample variability and the experiment repeatability. One sample was excluded from the study based on UniSp2 and UniSp6 analysis. The amplicon quality for each miRNA was assessed by analyzing the melting curves.

Hemolysis was determined using the cycle quantification value (Cq) difference between hsa-miR-23a-3p, which has been demonstrated to be unaffected by hemolysis, and hsa-miR-451a, which is highly expressed in red blood cells. Based on the results in the literature, we considered a difference lower than 7 Cq as acceptable [51, 52].

### Data processing

The Cq values were calculated by applying the second derivative method of the LightCycler 480 software version 1.5.0.

For the profiling study, we selected 184 miRNAs among the 372 used in the pilot study. The 184 miRNAs were selected according to the following criteria: 1) all miRNAs with a Cq lower than or equal to 36 cycles in at least one of the five groups investigated were considered for further analysis; 2) only miRNAs that showed an average Cq < 34 in at least one group or a Cq < 35 in at least two groups were analyzed. This method allowed the assessment of miRNAs that were present in all groups and those that were largely increased or decreased in either one specific group of patients or controls. For the analysis of the 184 miRNAs, the Cq value for all unamplified (lowly or unexpressed) miRNAs was set to a value of 40,in agreement with previous studies [53]. For normalization, the global mean normalization method (GMN) was applied to the data set. This method is particularly adapted for high-throughput miRNA profiling [54]. The fold-difference was calculated using the equation 2^- ΔΔCq^ [55].

For the validation study, 10 miRNAs were measured for 100 samples which were divided into 2 groups. In this case, the GMN method could not be applied because it is only validated for a large set of genes. Therefore, to minimize the variation due to differences in miRNA input, we proceeded with a quantification step of all miRNA samples [56]. A fixed amount of miRNAs was thus reverse transcribed. Then, to correct for technical variation, a ratio-based normalization approach was carried out by transforming the Cq values into arbitrary units and calculating an arithmetic mean of three endogenous controls (hsa-miR-16-5p, hsa-miR-106a-5p and hsa-miR-142-5p). Hsa-miR-16-5p and hsa-miR-106a-5p were selected as endogenous controls on the basis of a literature review [57–60]. Hsa-miR-142-5p was designated as the most stable miRNA among the pool of 184 miRNA candidates; these candidates were investigated during the profiling study using the NormFinder algorithm [61, 62].

### Statistical analysis

The differences between the groups were evaluated by the Wilcoxon-Mann-Whitney rank-sum test. The p-values were adjusted for multiple testing using the Benjamini-Hochberg correction rate. The analyses were performed with Stata 15.1 (StataCorp, College Station, TX, USA) and the R package [63].

Regarding the analysis of the profiling set, we selected miRNAs that provide the best prediction of the subject’s group using a random forest (RF) model. We aimed to 1) optimally classify participants into five groups, 2) assess the accuracy of such a classifier, and 3) select the most important miRNAs used by the classifier. RF, a very popular supervised machine learning algorithm [64], is a nonlinear and nonparametric method that also allows variable selection through variable importance measure (VIM). It is suitable for an initial screening analysis where the parameter/subject ratio is very high [65]. We used the cforest function provided in the R package Party [66]. Cforest is based on conditional inference trees [67] and is known to be robust to biases that may affect other RF implementations [68]. For VIM computation, we used the conditional variable importance method [69]; this method does not overstate the importance of correlated predictors and, hence, can be interpreted in a similar way as the coefficients of parametric regression models [70, 71].

In more detail, the procedure was as follows: 1) the predictors were the 184 miRNA expression levels; the outcome (group membership) was coded as a five-level categorical variable. 2) The cforest function was run with the parameters mtry = 20 and ntree = 1001; other parameters were set to default. This grew 1001 classification trees, each of them using a subset of 20 randomly selected predictors for splitting. 3) Using out-of-bag data, a confusion matrix was used to compare the true class (reference) with the class assigned by the classifier (prediction). The overall accuracy, as well as the sensitivity and specificity for each group, was also computed. Cohen’s kappa assessed the agreement between the prediction and the reference [72]. 4) The *varimp* function evaluated the variable importance of the 184 predictors (conditional importance method). We then computed the ‘mean decrease in accuracy’ importance scores of the 40 most important predictors. 5) To validate the importance scores, as suggested by Strobl et al. [71], we plotted a threshold which corresponded to the range of random fluctuations of irrelevant predictors around zero (informative predictors must exceed this limit).

To fulfill the second objective of our study, that is, differentiating nociceptive and neuropathic patients, we computed two supplemental RF models. For this step only patients (N = 73) were included. The goal was to identify one pain type (neuropathic or nociceptive) among the four pain types. In addition, we included only the 10 miRNAs identified with the highest VIM via the previous RF model. The codes for the dependent variable was one for the investigated pain type and zero for the others. The tuning parameters of the RF models were ntree = 1001 and mtry = 4. The out-of-bag estimates of the classification probabilities were computed for each patient. Based on those probabilities and the true classes, the receiver operating characteristic (ROC) curves were calculated along with the area under the curve (AUC) for assessing the predictive power of the selected miRNAs. The confidence intervals for the AUCs were obtained via bootstrapping. Finally, the VIM was computed to classify the miRNAs according to their importance for prediction.

For the validation analysis, a three-step analysis was conducted to identify the miRNAs that best differentiate between neuropathic and nociceptive pain in another sample of 100 patients. First, univariate analyses (Kruskal-Wallis one-way ANOVA on ranks) were run to select miRNAs that were influenced by the variable nociceptive/neuropathic pain. Then, all potential predictors were included in a first logistic regression analysis to determine the optimal number “N” of independent predictors. Finally, a second run of logistic regression with a forward selection of N predictors (with switching procedures) led to the best predictive model.

## Results

### Basic patient’s description

In the first round of the study (the profiling phase), we recruited eighty-four patients admitted to the Clinique romande de réadaptation and twenty-two controls (Ctrl) from the clinic’s staff and matched them for age, sex and body mass index (BMI). Twelve samples had to be removed due to hemoglobin contamination, which left 73 patients and 21 controls for analysis. The patients were attributed to 4 groups of chronic pain subtypes, i.e., 15 patients diagnosed with neuropathic pain (Np), 24 chronic patients with nociceptive pain (No), 17 patients with a combination of both nociceptive and neuropathic pain components (referred to as “mixed”), and 17 patients diagnosed with chronic regional pain syndrome (CRPS) (S1 Fig). There were no significant differences in age, sex or BMI between the 4 patient groups and the controls. The 4 groups of patients were also similar for all clinical data investigated in the profiling study.

For the second round (the validation phase) we recruited one hundred and nine patients diagnosed with chronic pain of either neuropathic or nociceptive origin. Nine samples were removed due to hemoglobin contamination and we thus ended up with 100 samples for analysis (40 Np and 60 No). Globally, patients did not significantly differ in their clinical features among these two groups. However, neuropathic patients exhibited a higher level of catastrophism when compared to nociceptive patients (p = 0.02) (Table 1).

We carefully monitored medication intake for the whole cohort of patients. Detailed description of prescribed analgesic can be found in S2 Table. As expected opioids and antiepileptic drugs were more used in the Np group compared to No (p<0.01). The most frequently prescribed non-analgesic medications were as follows: drugs for acid related disorders (A02), 20%; drugs to treat disorders of the cardiovascular system (C) 13%; antithrombotic agents (B01) 8%; lipid modifying agent (C10), 7%; with no statistical difference between groups.

### Phase 1: The profiling experiment for the screening of miRNA biomarkers

After processing the data obtained from the pilot phase, 184 miRNAs were selected based on their expression levels in the 5 different subject pools. On average, 180 miRNAs were detected per sample, of which 73% were expressed in all 94 subjects.

**Fig 2:**
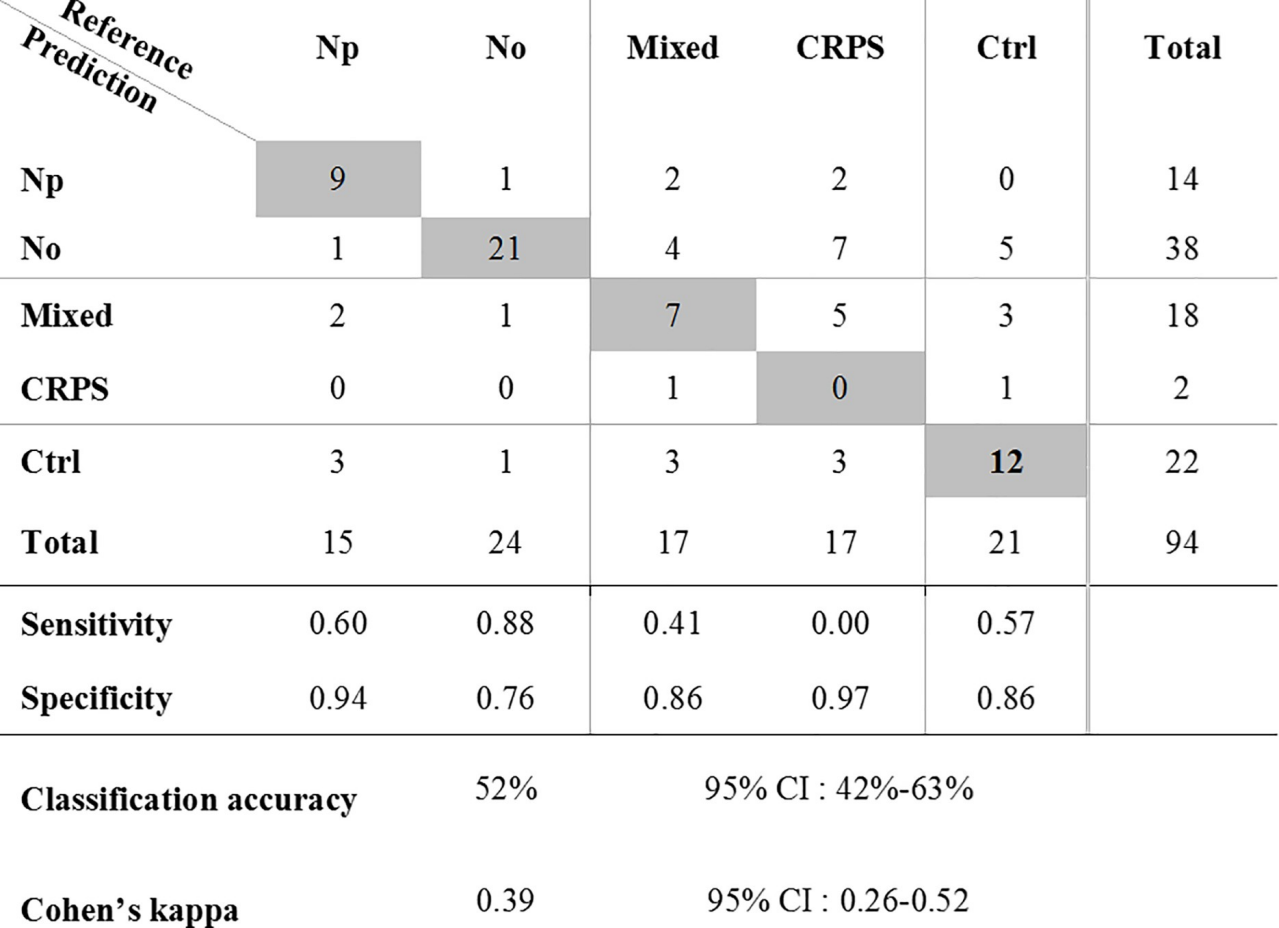
The confusion matrix for classifying the pain of 94 volunteers into five categories. The gray cells represent the number of patients correctly classified by the Random Forest model when compared to reference classification performed by the medical staff. Np: neuropathic, No: nociceptive, CRPS: chronic regional pain syndrome, Ctrl: control.

The RF model correctly classified half of the participants (52% accuracy) among the five groups. Furthermore, the discrimination power was moderate (kappa = 0.39). As shown in the confusion matrix (Fig 2), the CRPS and mixed patients were not adequately classified. In contrast, the neuropathic and nociceptive pain types were well separated from each other, with only 2 misclassified patients. When considering only these two groups of patients, the accuracy of the classification reaches 91% with a Cohen’s coefficient of 0.87 [0.63–1.11]_95%_. The RF model identifies the healthy controls reasonably well (57% sensitivity, 86% specificity).

The results of the first RF analysis suggest that the neuropathic and nociceptive pain types could be discriminated using 10 prominent miRNAs (Table 2). Theses miRNA were selected according to their VIM as previously described in the “Materials and Methods” section. The ROC curves (S2A and S2C Fig) indicate an AUC of 0.88 [0.67–0.96] _95%_ for the neuropathic group and 0.84 [0.72–0.92] _95%_ for the nociceptive group. VIM analyses (S2B and S2D Fig) show that 2–3 miRNAs were preferentially used for the discrimination. However, those miRNAs were different for the detection of neuropathic and nociceptive effects.

**Table 2:** miRBase accession number and the sequences of the 10 selected miRNAs.

Name	miRBase Accession number	Sequence
hsa-let-7d-5p	MI0000065	AGAGGUAGUAGGUUGCAUAGUU
hsa-miR-29c-3p	MI0000735	UAGCACCAUUUGAAAUCGGUUA
hsa-miR-98-5p	MI0000100	UGAGGUAGUAAGUUGUAUUGUU
hsa-miR-126-3p	MI0000471	UCGUACCGUGAGUAAUAAUGCG
hsa-miR-150-5p	MI0000479	UCUCCCAACCCUUGUACCAGUG
hsa-miR-205-5p	MI0000285	UCCUUCAUUCCACCGGAGUCUG
hsa-miR-222-3p	MI0000299	AGCUACAUCUGGCUACUGGGU
hsa-miR-320a	MI0000542	AAAAGCUGGGUUGAGAGGGCGA
hsa-miR-335-5p	MI0000816	UCAAGAGCAAUAACGAAAAAUGU
hsa-miR-423-5p	MI0001445	UGAGGGGCAGAGAGCGAGACUUU

### Phase 2: Validation of the miRNA candidates

**Fig 3:**
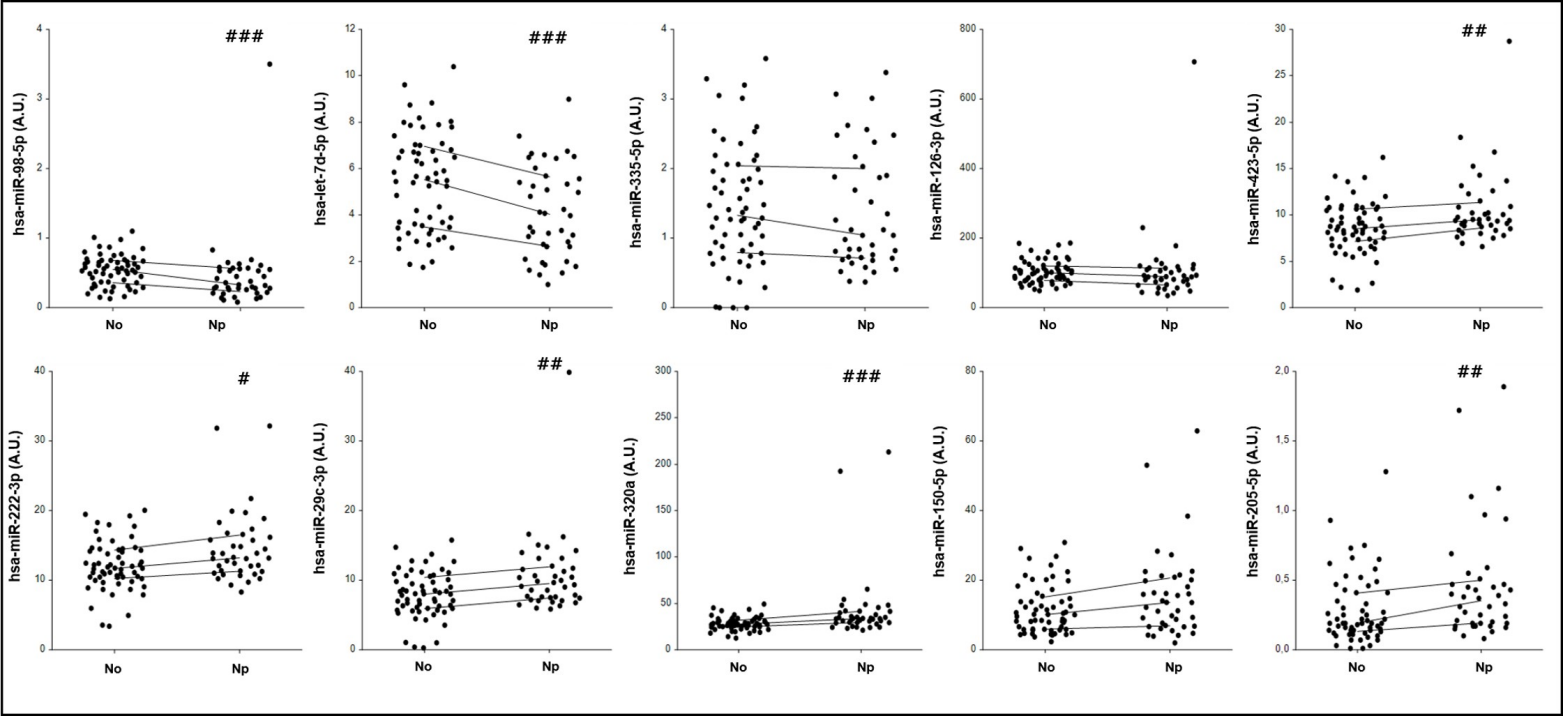
The differences in the miRNA expression levels between nociceptive (No) and neuropathic (Np) pain patients. Hsa-let-7d-5p, hsa-miR-205-5p, hsa-miR-222-3p, hsa-miR-29c-3p, hsa-miR-320a, hsa-miR-423-5p and hsa-miR-98-5p show significant differential expression between the nociceptive and neuropathic groups. Hsa-miR-126-3p, hsa-miR-150-5p and hsa-miR-335-5p were not significantly different between the two groups of patients. ^#^ p < 0.05; ^##^p < 0.01; ^###^p < 0.005.

For this phase, we finally included a total of 100 patients (40 Np and 60 No). From the 10 miRNAs selected after the profiling phase, the differential levels of miRNAs in neuropathic pain compared to the levels in the nociceptive pain subtype were validated for 7 of them using the Pick-and-Mix microRNA PCR Panel from Exiqon (Fig 3). The mRNA levels of hsa-miR-126-3p, hsa-miR-150-5p and hsa-miR-335-5p were not significantly different between the two groups of patients (Table 3).

**Table 3:** The validation phase fold differences in the expression levels determined by qRT-PCR for the representative 10 miRNAs differentiating nociceptive and neuropathic pain.

	Nociceptive			Neuropathic				
	Median	IQ 25%	IQ 75%	Median	IQ 25%	IQ 75%	Fold-difference	p-value
hsa-miR-98-5p	**0.55**	0.37	0.68	**0.33**	0.24	0.55	-1.67	**0.001**
hsa-let-7d-5p	**5.53**	3.51	6.91	**4.02**	2.63	5.62	-1.38	**0.003**
hsa-miR-335-5p	**1.33**	0.80	2.03	**1.04**	0.71	1.95	-1.28	0.43
hsa-miR-126-3p	**100.20**	79.23	118.82	**89.02**	66.26	113.03	-1.12	0.13
hsa-miR-423-5p	**8.55**	7.20	10.63	**9.46**	8.63	11.21	1.11	**0.009**
hsa-miR-222-3p	**11.72**	10.32	14.33	**13.23**	11.29	16.38	1.13	**0.012**
hsa-miR-29c-3p	**8.01**	5.94	10.32	**9.5**	7.47	11.88	1.19	**0.005**
hsa-miR-320a	**27.42**	24.42	31.50	**33.67**	29.32	41.48	1.23	**0.0001**
hsa-miR-150-5p	**10.11**	5.88	15.08	**13.61**	6.87	20.48	1.35	0.12
hsa-miR-205-5p	**0.19**	0.14	0.41	**0.35**	0.19	0.49	1.84	**0.008**

### Logistic regression

The univariable analyses confirmed that 7 miRNAs out of 10 could be influenced by the group and had a p-value < 0.05. As we usually include all the variables that reach a p-value up to 0.20 in the univariate tests in a regression model, we also considered hsa-miR-126-3p (p = 0.13) and hsa-miR-150-5p (p = 0.12). The logistic regression was then run with the 9 miRNAs (all but hsa-miR-335-5p, p = 0.43).

A preliminary multivariable regression analysis allowed reducing the size of the predictive model from a 9 to a 4, based on a very small increase in R2 beyond the 4 predictors (< 0.5%). Then, a second analysis was conducted to identify the best 4-size model. Hsa-miR-320a, hsa-miR-98-5p, hsa-miR-205-5p and hsa-miR-150-5p were selected as potential predictors in the model, but only the first two had significant regression coefficients (Wald probability level for miRNAs 320a/98/205/150: p = 0.001/0.024/0.178/0.244).

**Fig 4:**
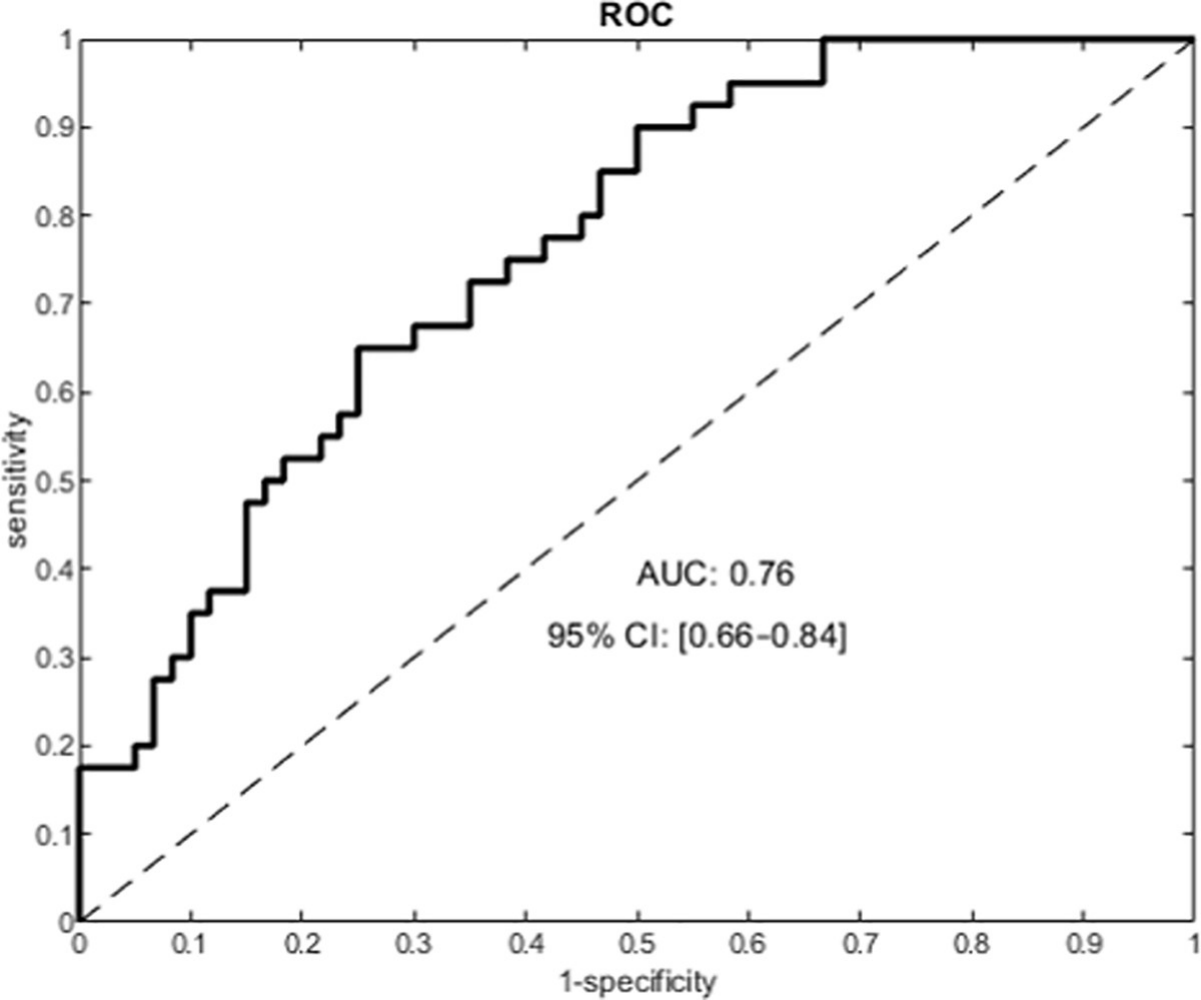
The receiver operating characteristics (ROC) curve built with the combination of 2 miRNAs (hsa-miR-320a and hsa-miR-98-5p) selected from the validation experiment. AUC: area under the curve; CI: confidence interval.

Finally, the 2-size model (hsa-miR-320a and hsa-miR-98-5p) was able to correctly classify 70 out of 100 patients. Using this model, 16% neuropathic patients were classified as nociceptive (false-negative), and 14% nociceptive patients were classified as neuropathic (false-positive). The AUC of the ROC curve was 0.77 (Fig 4)

## Discussion

Chronic pain represents a major health problem and affects one in five adults worldwide [73]. Its treatment requires improved detection and characterization tools. Chronic pain may be due to a long-term hyperexcitability of peripheral nociceptive and central neurons [74]. After nerve injury, hundreds of the genes and proteins that affect signal transduction or transmission are upregulated or down-regulated [75]. miRNAs are potent players in the control of protein expression. Their function in mature neurons is poorly investigated, but it has been demonstrated that they might contribute to mechanisms of synaptic plasticity [76]. In this context, c-miRNAs have been identified as promising biomarkers of chronic pain. In our study, we sought to investigate c-miRNA signatures in different types of chronic musculoskeletal pain. To our knowledge, this is the first study to compare plasma miRNA profiles in different subtypes of chronic musculoskeletal pain. From a panel of 184 miRNAs detectable in our plasma samples, we selected 10 c-miRNA candidates using a decision tree-based ensemble method. Interestingly, this selection of c-miRNAs was highly discriminatory for nociceptive and neuropathic pain etiology. After a validation phase, 7 out of these 10 c-miRNAs were differentially regulated in these two groups of patients.

In Table 3, we classified miRNA levels based on their fold-difference between nociceptive and neuropathic patients. When compared to the group of nociceptive pain, 2 of the c-miRNAs, hsa-miR-98-5p and hsa-let-7d-5p, were significantly down-regulated by 1.7- and 1.4-fold, respectively, in the neuropathic pain patients. Our fold-differences may appear to be relatively small. However, considering the heterogeneity of our subjects, the variations observed are in accordance with previous studies in the field showing fold-differences ranging from 0.45 to 2-folds [77] These two c-miRNAs are both Let-7 family members, and their expression levels were highly correlated in our study (Spearman’s rho = 0.67). As most of the miRNAs described so far in the literature, hsa-miR-98 and hsa-let-7d-5p have been mainly associated with different types of cancer. However, there is now evidence suggesting that these two miRNAs might play a role in the inflammation process associated with chronic pain. Thus, Bai et al. [78] demonstrated that the injection of complete Freund’s adjuvant (CFA) in the masseter muscle of rodents induced an upregulation of hsa-miR-98 when measured in the mandibular branch of the trigeminal nerve in the chronic phase of inflammation. These results are in accordance with our observations. Indeed, in chronic nociceptive patients where inflammation represents a major component, there is a higher level of hsa-miR-98-5p compared to that in the neuropathic group, which suggests a role for these miRNAs in the control of the inflammatory process.

Along the same lines of evidence, several Let-7 miRNA family members, including let-7d, have also been shown to play a key role in modulating the inflammatory response [79]. Specifically, hsa-let-7d-5p has been demonstrated to regulate the μ-opioid receptor signaling pathway, suggesting a potential role of this miRNA in the modulation of the effect of opioids [80]. Fibromyalgia syndrome, a chronic condition of widespread pain, has been extensively studied in connection with miRNA dysregulation. In a recent publication, Leinders et al. clearly demonstrated an association between the mean pain intensity and the hsa-let-7d-5p expression levels extracted from white blood cells [81].

When we focused on the miRNAs upregulated by neuropathic pain, 5 c-miRNAs were of particular interest. As previously discussed, the increase in the expression level of the c-miRNA observed in our study could be considered weak to moderate, ranging from 11 to 84%. Several of these miRNAs have been previously described in the literature and are associated with chronic pain. Thus, hsa-miR-320a has already been associated with different subtypes of musculoskeletal pain, such as CRPS of fibromyalgia [25]. Among other targets, hsa-miR-320a targets adenylate cyclase activating polypeptide 1 (ADCYAP1), a major regulator of neuropathic pain [82].

Moreover, the dysregulation of hsa-miR-29c has also been associated with CRPS [83] and neuropathic pain [84]. In a recent publication, Jeong et al. identified hsa-miR-29c as a critical factor involved in microglial activation and the development of neuropathic pain.

No direct link between hsa-miR-222 and pain has been reported thus far. However, a recent study demonstrated a potential role for these miRNAs in inhibiting apoptosis in neurons after a spinal cord injury [85]. These observations suggest that hsa-miR-222 may have a protective effect on neural cells after nerve injury.

Interestingly, our study revealed two miRNAs that were thus far not associated with chronic pain. First, hsa-miR-423 only showed a difference of 11% between the two groups. Hsa-miR-423-5p has been previously associated with myocardial infarction [86] and cancer [87]. However, the weak variation measured in our two groups of patients, despite its statistical significance, does not support a role of these miRNAs in the regulation or maintenance of chronic pain.

In contrast, hsa-miR-205 was increased by approximately 84% in the neuropathic pain patients when compared to the nociceptive group. Hsa-miR-205 has been broadly associated with different types of cancer. The analysis of the potential targets of hsa-miR-205 revealed an active role of these miRNAs in the control of transforming growth factor-β (TGF-β) signaling [88]. The latter, through its anti-inflammatory action, has been previously described as a potential therapeutic agent for neuropathic pain [89].

Thus, there are confirmations in the literature that some of our miRNA biomarkers are associated with chronic pain of musculoskeletal origin. However, our study is also the first to compare the miRNA profiles in nociceptive and neuropathic pain and to conclude significant differences in these two pain subtypes.

The reason why a tissue-specific dysregulation of miRNA expression associated with either nociceptive or neuropathic pain is observed is a matter of speculations. Much work needs to be done in order to elucidate if this increase is a cause or consequence of chronic pain. In a first attempt to answer this question, we are going to investigate if miRNA dysregulation occurs in a time dependent manner. We are currently, setting up a study with pain patients included in the early phase of their disease, shortly after the accident. As for the possible role of the dysregulated miRNAs, the most convincing approach would to use transgenic mice in animal models of pain.

Another important result of our study showed that the combination of 2 c-miRNAs (has-miR-320a and has-miR-98-5p) had a rather good predictive value for the discrimination between neuropathic and nociceptive origin of pain. Our model correctly classified 70% of the patients from the validation experiment. Despite the attention taken in our study to classify pain (multidisciplinary assessment, including the use of DN4 and assessment by a senior physician), we cannot ensure that all patients have been properly classified, as there is no gold standard for the diagnosis of neuropathic pain. This absence of a 100% reliable gold standard implies that in this kind of study, one should not expect to find excellent sensitivity and specificity indices, whatever the diagnostic method tested. This should encourage us to continue research on objective markers such as miRNAs that can help improve diagnostic procedures. Several other elements need to be taken into account in order to evaluate the quality of our model. First, as previously mentioned, we aimed to discriminate between two different types of chronic pain. Despite different origins of pain, these two populations of patients do not largely differ in their characteristics. In addition, the same neurotransmitters, neuropeptides, cytokines or enzymes are implicated in both types of pain with a large degree of overlap [90, 91]. These physiological similarities represent a major barrier for the development of an accurate biological test able to discriminate between these two types of pain. Another reason that may explain the lack of sensitivity of our diagnostic tool may come from the fact that our population of interest was very heterogeneous. To cover the majority of the patients admitted to our clinic, we deliberately chose the broadest inclusion criteria as possible. Previous studies have demonstrated that age or BMI, for instance, may influence miRNA expression [92]. Even if these two parameters did not significantly differ between the two groups of patients investigated in our validation study, we cannot exclude the possibility that this diversity in our patient panel may induce variability in miRNA expression and therefore reduce the predictability of our model. More stringent inclusion criteria would certainly have led to an improved prognostic model.

Questionnaires have been broadly used to discriminate neuropathic from non-neuropathic pain with certain success. Thus, the DN4 questionnaire has been shown to display excellent sensitivity and specificity, 83% and 90%, respectively, in various syndromes of neuropathic pain (i.e., painful diabetic neuropathy, spinal cord injury, cancer…). However, weaker discriminative properties (62% sensitivity, 44% specificity) were also reported for this questionnaire in specific pain conditions, such as lower back or neck pain [93]. The level of inaccurate diagnoses (i.e., overdiagnosis or underdiagnosis) obtained from screening questionnaires may be observed in 10 to 20% of the cases in the general population and in specific medical conditions such as widespread pain [94]. Moreover, questionnaires can also miss potentially relevant descriptors of neuropathic pain such as numbness or pain induced by heat that have not been reported in the Neuropathic Pain Symptom Inventory [29]. In this context, the development of an accurate biological test would be rather useful.

The wrong diagnosis of neuropathic pain in a patient is damaging because the specific treatment takes place over a period of at least several weeks, with side effects that disturb everyday life (particularly sedative effects) [95]. On the other hand, failure to diagnose neuropathic pain is less deleterious because the ineffectiveness of nociceptive analgesic treatment can be quickly confirmed. In this case, the advice of a specialist may be sought.

The use of scores such as DN4 remains the best diagnostic test for the detection of neuropathic pain by the nonspecialist physician. In our cohort, the false positive rate for DN4 was 12%, and the false negative rate was 2%. Based on the DN4-miRNA association, the false-positive and false-negative rates were both at 0% when the two tests were consistent (64% of cases). If this result was confirmed in a larger cohort, it would give the general practitioner a quick and inexpensive tool for screening for neuropathic pain. The specialist should then only be called upon in the event of a discrepancy between the 2 tests and in the event of a failure of the first treatment line. On a larger scale, a medical-economic study is needed to develop such a decision tree.

## Limitations

The present cross-sectional study does not allow us to exclude the possibility that the regulation of miRNA expression levels may evolve over time. Indeed, it is well known that chronic pain is associated with several other health-related disorders that might appear in a time-dependent manner (i.e., anxiety, depression …) and could influence the miRNA expression patterns. It would be of prime importance to assess, in a longitudinal study, the possible temporal changes in miRNA expression linked with the process of pain chronicization. Another limitation of our work relies on the choice of the miRNAs investigated. Indeed, as previously described, we decided to focus on a pool of 372 c-miRNAs that have been already described in the literature. According to a recent study published by Alles et al. the total number of human mature miRNA should be around 2300 [13]. However, information about the role or function of most of these miRNA candidates remains incomplete. Therefore, we decided to focus only on the best described ones. Once again, we cannot completely exclude the possibility that important miRNAs not included in our analysis could be pertinent biomarkers of chronic pain. Finally, as expected Np patients used more opioid and antiepileptic treatments than No. Since it is known that some treatments may influence miRNAs expression [34, 35] analgesic treatment could be a confounding bias in the relationship between the type of pain and the expression of miRNAs. The only ethically acceptable way to rule out this hypothesis would be to conduct a longitudinal observational study on a new cohort of chronic pain patients, and to observe the evolution of c-miRNAs during the course of the respective treatments.

## Conclusions

The results of this study demonstrate that some c-miRNAs are regulated differently in two distinct subtypes of chronic pain. This study confirmed the potential of these molecules to be biomarkers for the determination of the origin of chronic pain. At this stage, further work would be needed to determine why certain c-miRNAs are specifically modulated in the context of neuropathic pain while others are modulated in the context of nociceptive pain.

## Supporting information

S1 Table A list of the 184 miRNAs tested in the profiling phase. (DOCX)

S2 Table List and repartition of analgesic medication among the different groups of patients (sorted by ATC code). (DOCX)

S1 Fig Flow chart of the study. (TIF)

S2 Fig The combination of 10 miRNAs from the profiling experiment clearly distinguished nociceptive and neuropathic pain. The receiver operating characteristics (ROC) and the variable importance measure (VIM) of 10 miRNAs in patients with neuropathic (A and B) and nociceptive (C and D) pain. AUC: area under the curve; CI: confidence interval.
(TIF)

## Abbreviations

miRNAs: microRNAs
c-miRNAs: circulating microRNAs
No: nociceptive pain
Np: neuropathic pain
CRPS: chronic regional pain syndrome
mRNAs: messenger RNAs
CMSP: chronic musculoskeletal pain
DN4: Douleur Neuropathique en 4 Questions
Ctrl: control
BPI: Brief Pain Inventory
HADS: Hospital Anxiety and Depression Scale
TSK: Tampa Scale for Kinesiophobia
PCS: Pain Catastrophizing Scale
CIRS: Cumulative Illness Rating Scale
EDTA: ethylenediaminetetraacetic acid
RNA: ribonucleic acid
cDNA: complementary deoxyribonucleic acid
RT: reverse transcription
PCR: polymerase chain reaction
CV: coefficient of variation
Cq: quantification value
GMN: global mean normalization
RF: random forest
VIM: variable importance measure
IQR: interquartile range
ROC: receiver operating characteristic
AUC: area under the curve
BMI: body mass index
CFA: Complete Freund’s adjuvant
