# Correction: Differences in the miRNA signatures of chronic musculoskeletal pain patients from neuropathic or nociceptive origins

**DOI:** 10.1371/journal.pone.0220486

**Published:** 2019-07-25

**Authors:** The PLOS ONE Staff

## Notice of republication

An incorrect version of Fig 3 was published in error. The publisher apologizes for this error. This article was republished on July 15, 2019 to correct for this error. Please download this article again to view the correct version.
