# Supplementary material for: Differences in the miRNA signatures of chronic musculoskeletal pain patients from neuropathic or nociceptive origins
Source: PLoS One. 2019 Jul 5;14(7):e0219311. doi: 10.1371/journal.pone.0219311 (PMC6611606; doi:10.1371/journal.pone.0219311)
Supplement: S1 Table — (DOCX) [file pone.0219311.s001.docx]

| microRNA Name | Target sequence | Exiqon LNA™ PCR primer set, Product No | Corresponding Qiagen Product No |
| --- | --- | --- | --- |
| hsa-let-7a-5p | UGAGGUAGUAGGUUGUAUAGUU | 205727 | YP00205727 |
| hsa-let-7b-5p | UGAGGUAGUAGGUUGUGUGGUU | 204750 | YP00204750 |
| hsa-let-7c-5p | UGAGGUAGUAGGUUGUAUGGUU | 204767 | YP00204767 |
| hsa-let-7d-3p | CUAUACGACCUGCUGCCUUUCU | 205627 | YP00205627 |
| hsa-let-7d-5p | AGAGGUAGUAGGUUGCAUAGUU | 204124 | YP00204124 |
| hsa-let-7e-5p | UGAGGUAGGAGGUUGUAUAGUU | 205711 | YP00205711 |
| hsa-let-7f-5p | UGAGGUAGUAGAUUGUAUAGUU | 204359 | YP00204359 |
| hsa-let-7g-5p | UGAGGUAGUAGUUUGUACAGUU | 204565 | YP00204565 |
| hsa-let-7i-5p | UGAGGUAGUAGUUUGUGCUGUU | 204394 | YP00204394 |
| hsa-miR-1 | UGGAAUGUAAAGAAGUAUGUAU | 204344 | YP00204344 |
| hsa-miR-100-5p | AACCCGUAGAUCCGAACUUGUG | 205689 | YP00205689 |
| hsa-miR-101-3p | UACAGUACUGUGAUAACUGAA | 204786 | YP00204786 |
| hsa-miR-103a-3p | AGCAGCAUUGUACAGGGCUAUGA | 204063 | YP00204063 |
| hsa-miR-106a-5p | AAAAGUGCUUACAGUGCAGGUAG | 204563 | YP00204563 |
| hsa-miR-106b-5p | UAAAGUGCUGACAGUGCAGAU | 205884 | YP00205884 |
| hsa-miR-107 | AGCAGCAUUGUACAGGGCUAUCA | 204468 | YP00204468 |
| hsa-miR-122-5p | UGGAGUGUGACAAUGGUGUUUG | 205664 | YP00205664 |
| hsa-miR-125a-5p | UCCCUGAGACCCUUUAACCUGUGA | 204339 | YP00204339 |
| hsa-miR-125b-5p | UCCCUGAGACCCUAACUUGUGA | 205713 | YP00205713 |
| hsa-miR-126-3p | UCGUACCGUGAGUAAUAAUGCG | 204227 | YP00204227 |
| hsa-miR-126-5p | CAUUAUUACUUUUGGUACGCG | 206010 | YP00206010 |
| hsa-miR-127-3p | UCGGAUCCGUCUGAGCUUGGCU | 204048 | YP00204048 |
| hsa-miR-128-3p | UCACAGUGAACCGGUCUCUUU | 205995 | YP00205995 |
| hsa-miR-130a-3p | CAGUGCAAUGUUAAAAGGGCAU | 204658 | YP00204658 |
| hsa-miR-130b-3p | CAGUGCAAUGAUGAAAGGGCAU | 204317 | YP00204317 |
| hsa-miR-132-3p | UAACAGUCUACAGCCAUGGUCG | 206035 | YP00206035 |
| hsa-miR-133a-3p | UUUGGUCCCCUUCAACCAGCUG | 204788 | YP00204788 |
| hsa-miR-133b | UUUGGUCCCCUUCAACCAGCUA | 206058 | YP00206058 |
| hsa-miR-134-5p | UGUGACUGGUUGACCAGAGGGG | 205989 | YP00205989 |
| hsa-miR-136-5p | ACUCCAUUUGUUUUGAUGAUGGA | 204779 | YP00204779 |
| hsa-miR-139-5p | UCUACAGUGCACGUGUCUCCAGU | 205874 | YP00205874 |
| hsa-miR-140-3p | UACCACAGGGUAGAACCACGG | 204304 | YP00204304 |
| hsa-miR-140-5p | CAGUGGUUUUACCCUAUGGUAG | 204540 | YP00204540 |
| hsa-miR-141-3p | UAACACUGUCUGGUAAAGAUGG | 204504 | YP00204504 |
| hsa-miR-142-3p | UGUAGUGUUUCCUACUUUAUGGA | 204291 | YP00204291 |
| hsa-miR-142-5p | CAUAAAGUAGAAAGCACUACU | 204722 | YP00204722 |
| hsa-miR-143-3p | UGAGAUGAAGCACUGUAGCUC | 205992 | YP00205992 |
| hsa-miR-144-3p | UACAGUAUAGAUGAUGUACU | 204754 | YP00204754 |
| hsa-miR-145-5p | GUCCAGUUUUCCCAGGAAUCCCU | 204483 | YP00204483 |
| hsa-miR-146a-5p | UGAGAACUGAAUUCCAUGGGUU | 204688 | YP00204688 |
| hsa-miR-148a-3p | UCAGUGCACUACAGAACUUUGU | 205867 | YP00205867 |
| hsa-miR-148b-3p | UCAGUGCAUCACAGAACUUUGU | 204047 | YP00204047 |
| hsa-miR-150-5p | UCUCCCAACCCUUGUACCAGUG | 204660 | YP00204660 |
| hsa-miR-151a-3p | CUAGACUGAAGCUCCUUGAGG | 204576 | YP00204576 |
| hsa-miR-151a-5p | UCGAGGAGCUCACAGUCUAGU | 204007 | YP00204007 |
| hsa-miR-152-3p | UCAGUGCAUGACAGAACUUGG | 204294 | YP00204294 |
| hsa-miR-154-5p | UAGGUUAUCCGUGUUGCCUUCG | 204518 | YP00204518 |
| hsa-miR-155-5p | UUAAUGCUAAUCGUGAUAGGGGU | 204308 | YP00204308 |
| hsa-miR-15a-5p | UAGCAGCACAUAAUGGUUUGUG | 204066 | YP00204066 |
| hsa-miR-15b-5p | UAGCAGCACAUCAUGGUUUACA | 204243 | YP00204243 |
| hsa-miR-16-5p | UAGCAGCACGUAAAUAUUGGCG | 205702 | YP00205702 |
| hsa-miR-17-5p | CAAAGUGCUUACAGUGCAGGUAG | 204771 | YP02119304 |
| hsa-miR-181a-5p | AACAUUCAACGCUGUCGGUGAGU | 206081 | YP00206081 |
| hsa-miR-185-5p | UGGAGAGAAAGGCAGUUCCUGA | 206037 | YP00206037 |
| hsa-miR-186-5p | CAAAGAAUUCUCCUUUUGGGCU | 206053 | YP00206053 |
| hsa-miR-18a-5p | UAAGGUGCAUCUAGUGCAGAUAG | 204207 | YP00204207 |
| hsa-miR-18b-5p | UAAGGUGCAUCUAGUGCAGUUAG | 204084 | YP00204084 |
| hsa-miR-191-5p | CAACGGAAUCCCAAAAGCAGCUG | 204306 | YP00204306 |
| hsa-miR-192-5p | CUGACCUAUGAAUUGACAGCC | 204099 | YP00204099 |
| hsa-miR-193b-3p | AACUGGCCCUCAAAGUCCCGCU | 204226 | YP00204226 |
| hsa-miR-194-5p | UGUAACAGCAACUCCAUGUGGA | 204080 | YP00204080 |
| hsa-miR-197-3p | UUCACCACCUUCUCCACCCAGC | 204380 | YP00204380 |
| hsa-miR-199a-3p | ACAGUAGUCUGCACAUUGGUUA | 204536 | YP00204536 |
| hsa-miR-199a-5p | CCCAGUGUUCAGACUACCUGUUC | 204494 | YP00204494 |
| hsa-miR-19a-3p | UGUGCAAAUCUAUGCAAAACUGA | 205862 | YP00205862 |
| hsa-miR-19b-3p | UGUGCAAAUCCAUGCAAAACUGA | 204450 | YP00204450 |
| hsa-miR-200b-3p | UAAUACUGCCUGGUAAUGAUGA | 206071 | YP00206071 |
| hsa-miR-200c-3p | UAAUACUGCCGGGUAAUGAUGGA | 204482 | YP00204482 |
| hsa-miR-205-5p | UCCUUCAUUCCACCGGAGUCUG | 204487 | YP00204487 |
| hsa-miR-20a-5p | UAAAGUGCUUAUAGUGCAGGUAG | 204292 | YP00204292 |
| hsa-miR-210-3p | CUGUGCGUGUGACAGCGGCUGA | 204333 | YP00204333 |
| hsa-miR-215-5p | AUGACCUAUGAAUUGACAGAC | 204598 | YP00204598 |
| hsa-miR-21-5p | UAGCUUAUCAGACUGAUGUUGA | 204230 | YP00204230 |
| hsa-miR-221-3p | AGCUACAUUGUCUGCUGGGUUUC | 204532 | YP00204532 |
| hsa-miR-222-3p | AGCUACAUCUGGCUACUGGGU | 204551 | YP00204551 |
| hsa-miR-223-3p | UGUCAGUUUGUCAAAUACCCCA | 205986 | YP00205986 |
| hsa-miR-22-3p | AAGCUGCCAGUUGAAGAACUGU | 204606 | YP00204606 |
| hsa-miR-22-5p | AGUUCUUCAGUGGCAAGCUUUA | 204255 | YP00204255 |
| hsa-miR-23a-3p | AUCACAUUGCCAGGGAUUUCC | 204772 | YP00204772 |
| hsa-miR-23b-3p | AUCACAUUGCCAGGGAUUACC | 204790 | YP00204790 |
| hsa-miR-24-3p | UGGCUCAGUUCAGCAGGAACAG | 204260 | YP00204260 |
| hsa-miR-25-3p | CAUUGCACUUGUCUCGGUCUGA | 204361 | YP00204361 |
| hsa-miR-26a-5p | UUCAAGUAAUCCAGGAUAGGCU | 206023 | YP00206023 |
| hsa-miR-26b-5p | UUCAAGUAAUUCAGGAUAGGU | 204172 | YP00204172 |
| hsa-miR-27a-3p | UUCACAGUGGCUAAGUUCCGC | 206038 | YP00206038 |
| hsa-miR-27b-3p | UUCACAGUGGCUAAGUUCUGC | 205915 | YP00205915 |
| hsa-miR-28-5p | AAGGAGCUCACAGUCUAUUGAG | 204322 | YP00204322 |
| hsa-miR-296-5p | AGGGCCCCCCCUCAAUCCUGU | 204436 | YP00204436 |
| hsa-miR-299-5p | UGGUUUACCGUCCCACAUACAU | 204544 | YP00204544 |
| hsa-miR-29a-3p | UAGCACCAUCUGAAAUCGGUUA | 204698 | YP00204698 |
| hsa-miR-29b-3p | UAGCACCAUUUGAAAUCAGUGUU | 204679 | YP00204679 |
| hsa-miR-29c-3p | UAGCACCAUUUGAAAUCGGUUA | 204729 | YP00204729 |
| hsa-miR-301a-3p | CAGUGCAAUAGUAUUGUCAAAGC | 205601 | YP00205601 |
| hsa-miR-301b | CAGUGCAAUGAUAUUGUCAAAGC | 204390 | YP00204390 |
| hsa-miR-30a-5p | UGUAAACAUCCUCGACUGGAAG | 205695 | YP00205695 |
| hsa-miR-30b-5p | UGUAAACAUCCUACACUCAGCU | 204765 | YP00204765 |
| hsa-miR-30c-5p | UGUAAACAUCCUACACUCUCAGC | 204783 | YP00204783 |
| hsa-miR-30d-5p | UGUAAACAUCCCCGACUGGAAG | 206047 | YP00206047 |
| hsa-miR-30e-3p | CUUUCAGUCGGAUGUUUACAGC | 204410 | YP00204410 |
| hsa-miR-30e-5p | UGUAAACAUCCUUGACUGGAAG | 204714 | YP00204714 |
| hsa-miR-320a | AAAAGCUGGGUUGAGAGGGCGA | 206042 | YP00206042 |
| hsa-miR-323a-3p | CACAUUACACGGUCGACCUCU | 204278 | YP00204278 |
| hsa-miR-324-3p | ACUGCCCCAGGUGCUGCUGG | 204303 | YP00204303 |
| hsa-miR-324-5p | CGCAUCCCCUAGGGCAUUGGUGU | 204057 | YP00204057 |
| hsa-miR-32-5p | UAUUGCACAUUACUAAGUUGCA | 204792 | YP00204792 |
| hsa-miR-326 | CCUCUGGGCCCUUCCUCCAG | 204512 | YP00204512 |
| hsa-miR-328-3p | CUGGCCCUCUCUGCCCUUCCGU | 204364 | YP00204364 |
| hsa-miR-329-3p | AACACACCUGGUUAACCUCUUU | 206052 | YP00206052 |
| hsa-miR-330-3p | GCAAAGCACACGGCCUGCAGAGA | 204017 | YP00204017 |
| hsa-miR-331-3p | GCCCCUGGGCCUAUCCUAGAA | 206046 | YP00206046 |
| hsa-miR-335-5p | UCAAGAGCAAUAACGAAAAAUGU | 204151 | YP02119293 |
| hsa-miR-337-3p | CUCCUAUAUGAUGCCUUUCUUC | 205938 | YP00205938 |
| hsa-miR-338-3p | UCCAGCAUCAGUGAUUUUGUUG | 204719 | YP00204719 |
| hsa-miR-339-5p | UCCCUGUCCUCCAGGAGCUCACG | 206007 | YP00206007 |
| hsa-miR-33a-5p | GUGCAUUGUAGUUGCAUUGCA | 205690 | YP00205690 |
| hsa-miR-340-5p | UUAUAAAGCAAUGAGACUGAUU | 206068 | YP00206068 |
| hsa-miR-342-3p | UCUCACACAGAAAUCGCACCCGU | 205625 | YP00205625 |
| hsa-miR-345-5p | GCUGACUCCUAGUCCAGGGCUC | 206006 | YP00206006 |
| hsa-miR-34a-5p | UGGCAGUGUCUUAGCUGGUUGU | 204486 | YP00204486 |
| hsa-miR-361-5p | UUAUCAGAAUCUCCAGGGGUAC | 206054 | YP00206054 |
| hsa-miR-363-3p | AAUUGCACGGUAUCCAUCUGUA | 204726 | YP00204726 |
| hsa-miR-365a-3p | UAAUGCCCCUAAAAAUCCUUAU | 204622 | YP00204622 |
| hsa-miR-370-3p | GCCUGCUGGGGUGGAACCUGGU | 204011 | YP00204011 |
| hsa-miR-374a-5p | UUAUAAUACAACCUGAUAAGUG | 204758 | YP00204758 |
| hsa-miR-374b-5p | AUAUAAUACAACCUGCUAAGUG | 204608 | YP00204608 |
| hsa-miR-375 | UUUGUUCGUUCGGCUCGCGUGA | 204362 | YP00204362 |
| hsa-miR-376a-3p | AUCAUAGAGGAAAAUCCACGU | 204508 | YP00204508 |
| hsa-miR-376b-3p | AUCAUAGAGGAAAAUCCAUGUU | 204218 | YP00204218 |
| hsa-miR-376c-3p | AACAUAGAGGAAAUUCCACGU | 204442 | YP00204442 |
| hsa-miR-377-3p | AUCACACAAAGGCAACUUUUGU | 204733 | YP00204733 |
| hsa-miR-379-5p | UGGUAGACUAUGGAACGUAGG | 205658 | YP00205658 |
| hsa-miR-382-5p | GAAGUUGUUCGUGGUGGAUUCG | 204169 | YP00204169 |
| hsa-miR-409-3p | GAAUGUUGCUCGGUGAACCCCU | 204358 | YP00204358 |
| hsa-miR-410-3p | AAUAUAACACAGAUGGCCUGU | 204042 | YP00204042 |
| hsa-miR-411-5p | UAGUAGACCGUAUAGCGUACG | 204531 | YP00204531 |
| hsa-miR-421 | AUCAACAGACAUUAAUUGGGCGC | 204603 | YP00204603 |
| hsa-miR-423-3p | AGCUCGGUCUGAGGCCCCUCAGU | 204488 | YP00204488 |
| hsa-miR-423-5p | UGAGGGGCAGAGAGCGAGACUUU | 205624 | YP00205624 |
| hsa-miR-424-5p | CAGCAGCAAUUCAUGUUUUGAA | 204736 | YP00204736 |
| hsa-miR-425-3p | AUCGGGAAUGUCGUGUCCGCCC | 204038 | YP00204038 |
| hsa-miR-425-5p | AAUGACACGAUCACUCCCGUUGA | 204337 | YP00204337 |
| hsa-miR-431-5p | UGUCUUGCAGGCCGUCAUGCA | 204737 | YP00204737 |
| hsa-miR-432-5p | UCUUGGAGUAGGUCAUUGGGUGG | 204776 | YP00204776 |
| hsa-miR-433-3p | AUCAUGAUGGGCUCCUCGGUGU | 204036 | YP00204036 |
| hsa-miR-451a | AAACCGUUACCAUUACUGAGUU | 204734 | YP02119305 |
| hsa-miR-454-3p | UAGUGCAAUAUUGCUUAUAGGGU | 205663 | YP00205663 |
| hsa-miR-484 | UCAGGCUCAGUCCCCUCCCGAU | 205636 | YP00205636 |
| hsa-miR-485-3p | GUCAUACACGGCUCUCCUCUCU | 206055 | YP00206055 |
| hsa-miR-486-5p | UCCUGUACUGAGCUGCCCCGAG | 204001 | YP00204001 |
| hsa-miR-487b-3p | AAUCGUACAGGGUCAUCCACUU | 204489 | YP00204489 |
| hsa-miR-491-5p | AGUGGGGAACCCUUCCAUGAGG | 204695 | YP00204695 |
| hsa-miR-495-3p | AAACAAACAUGGUGCACUUCUU | 206015 | YP00206015 |
| hsa-miR-497-5p | CAGCAGCACACUGUGGUUUGU | 204354 | YP00204354 |
| hsa-miR-503-5p | UAGCAGCGGGAACAGUUCUGCAG | 204334 | YP00204334 |
| hsa-miR-505-3p | CGUCAACACUUGCUGGUUUCCU | 204214 | YP00204214 |
| hsa-miR-532-3p | CCUCCCACACCCAAGGCUUGCA | 204003 | YP00204003 |
| hsa-miR-532-5p | CAUGCCUUGAGUGUAGGACCGU | 204221 | YP00204221 |
| hsa-miR-539-5p | GGAGAAAUUAUCCUUGGUGUGU | 205656 | YP00205656 |
| hsa-miR-545-3p | UCAGCAAACAUUUAUUGUGUGC | 206087 | YP00206087 |
| hsa-miR-551b-3p | GCGACCCAUACUUGGUUUCAG | 204067 | YP00204067 |
| hsa-miR-574-3p | CACGCUCAUGCACACACCCACA | 206011 | YP00206011 |
| hsa-miR-584-5p | UUAUGGUUUGCCUGGGACUGAG | 204568 | YP00204568 |
| hsa-miR-590-5p | GAGCUUAUUCAUAAAAGUGCAG | 204222 | YP00204222 |
| hsa-miR-598-3p | UACGUCAUCGUUGUCAUCGUCA | 204320 | YP00204320 |
| hsa-miR-625-3p | GACUAUAGAACUUUCCCCCUCA | 204647 | YP00204647 |
| hsa-miR-628-3p | UCUAGUAAGAGUGGCAGUCGA | 206057 | YP00206057 |
| hsa-miR-629-5p | UGGGUUUACGUUGGGAGAACU | 204370 | YP00204370 |
| hsa-miR-652-3p | AAUGGCGCCACUAGGGUUGUG | 204387 | YP00204387 |
| hsa-miR-654-5p | UGGUGGGCCGCAGAACAUGUGC | 204439 | YP00204439 |
| hsa-miR-660-5p | UACCCAUUGCAUAUCGGAGUUG | 205911 | YP00205911 |
| hsa-miR-744-5p | UGCGGGGCUAGGGCUAACAGCA | 204663 | YP00204663 |
| hsa-miR-766-3p | ACUCCAGCCCCACAGCCUCAGC | 204499 | YP00204499 |
| hsa-miR-874-3p | CUGCCCUGGCCCGAGGGACCGA | 204761 | YP00204761 |
| hsa-miR-877-5p | GUAGAGGAGAUGGCGCAGGG | 205626 | YP00205626 |
| hsa-miR-885-5p | UCCAUUACACUACCCUGCCUCU | 204473 | YP00204473 |
| hsa-miR-92a-3p | UAUUGCACUUGUCCCGGCCUGU | 204258 | YP00204258 |
| hsa-miR-93-5p | CAAAGUGCUGUUCGUGCAGGUAG | 204715 | YP00204715 |
| hsa-miR-940 | AAGGCAGGGCCCCCGCUCCCC | 204094 | YP00204094 |
| hsa-miR-98-5p | UGAGGUAGUAAGUUGUAUUGUU | 204640 | YP00204640 |
| hsa-miR-99a-5p | AACCCGUAGAUCCGAUCUUGUG | 204521 | YP00204521 |
| hsa-miR-99b-5p | CACCCGUAGAACCGACCUUGCG | 205983 | YP00205983 |
| mmu-miR-378a-3p | ACUGGACUUGGAGUCAGAAGG | 204179 | YP00204179 |
| SNORD38B |  | 203901 | YP00203901 |
| U6 snRNA |  | 203907 | YP00203907 |
