# Supplementary material for: Differences in the miRNA signatures of chronic musculoskeletal pain patients from neuropathic or nociceptive origins
Source: PLoS One. 2019 Jul 5;14(7):e0219311. doi: 10.1371/journal.pone.0219311 (PMC6611606; doi:10.1371/journal.pone.0219311)
Supplement: S2 Table — (DOCX) [file pone.0219311.s002.docx]

|  | **Profiling set** | | | | | **Validation set** | | |
| --- | --- | --- | --- | --- | --- | --- | --- | --- |
| **Family (ATC Code)** | **Np** (N=15) | **No** (N=24) | **Mixed** (N=17) | **CRPS** (N=17) | **p-values** | **Np** (N=40) | **No** (N=60) | **p-values** |
| Nonsteroidal anti-inflammatory drugs (M01A) | 3 (21%) | 6 (25%) | 2 (12%) | 3 (18%) | 0.76 | 8 (20%) | 14 (23%) | 0.69 |
| Opioids (N02A) | 5 (36%) | 6 (25%) | 6 (35%) | 5 (29%) | 0.87 | 18 (45%) | 13 (22%) | 0.01 |
| Analgesics and antipyretics (N02B) | 2 (14%) | 4 (17%) | 4 (24%) | 7 (41%) | 0.24 | 12 (30%) | 14 (23%) | 0.46 |
| Antiepileptics (N03A) | 8 (57%) | 2 (8%) | 5 (29%) | 5 (29%) | 0.01 | 17 (43%) | 2 (3%) | <0.01 |
| Antidepressant (N06A) | 3 (21%) | 5 (21%) | 2 (12%) | 5 (29%) | 0.66 | 11 (28%) | 8 (13%) | 0.08 |
