# Supplementary figures and images for: Differences in the miRNA signatures of chronic musculoskeletal pain patients from neuropathic or nociceptive origins
Source: PLoS One. 2019 Jul 5;14(7):e0219311. doi: 10.1371/journal.pone.0219311 (PMC6611606; doi:10.1371/journal.pone.0219311)

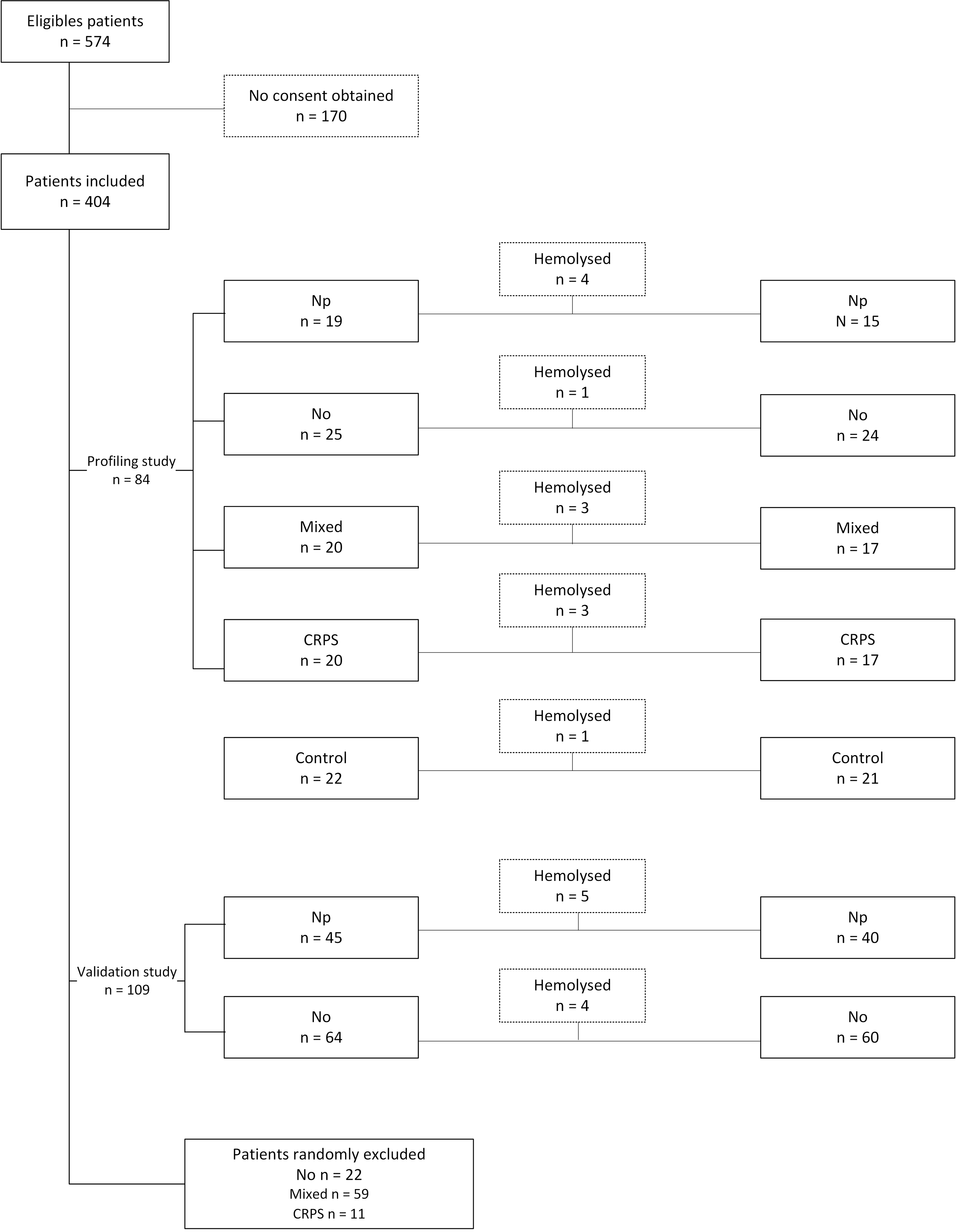

Supplement: S1 Fig — (TIF) [file pone.0219311.s003.tif]

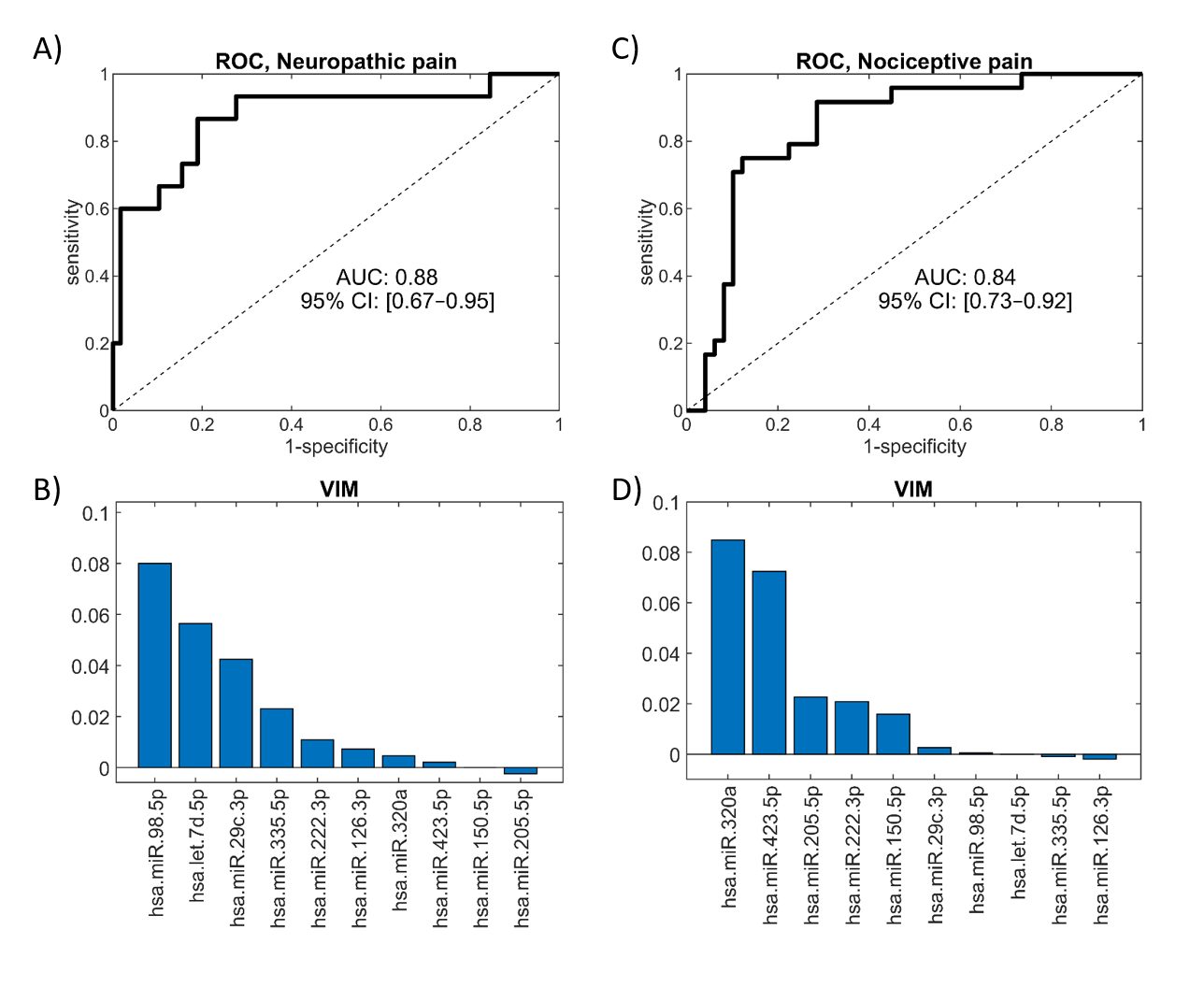

Supplement: S2 Fig — The receiver operating characteristics (ROC) and the variable importance measure (VIM) of 10 miRNAs in patients with neuropathic (A and B) and nociceptive (C and D) pain. AUC: area under the curve; CI: confidence interval. (TIF) [file pone.0219311.s004.tif]
